# Meta-analysis on last ten years of clinical injection of bone marrow-derived and umbilical cord MSC to reverse cirrhosis or rescue patients with acute-on-chronic liver failure

**DOI:** 10.1186/s13287-023-03494-2

**Published:** 2023-09-23

**Authors:** Huimin Wang, Weiqi Yao, Yuyan Wang, Haibo Dong, Tengyun Dong, Wangyi Zhou, Lingling Cui, Lulu Zhao, Yu Zhang, Lei Shi, Yingan Jiang

**Affiliations:** 1 Department of Infectious Diseases, Renmin Hospital of Wuhan University, No. 99 Zhangzhidong Road, Wuchang District, Hubei, China; 2 Wuhan Optics Valley Vcanbiopharma Co., Ltd., Hubei, China; 3 Key Industrial Base for Stem Cell Engineering Products, No. 12 Meiyuan Road, Tianjin, China; 4 Department of Biology and Medicine, Hubei University of Technology, Wuhan, China; 5 Department of Laboratory Medicine, The First Affiliated Hospital of Yangtze University, Jingzhou, China; 6 Department of Physiology and Pathophysiology, Tianjin Medical University, Tianjin, China; 7 Department of Infectious Diseases, Fifth Medical Center of Chinese, Fengtai District, PLA General Hospital, National Clinical Research Center for Infectious Diseases, No. 100 Xi Si Huan Middle Road, Beijing, China

**Keywords:** Mesenchymal stem cells, Decompensated liver cirrhosis, Acute-on-chronic liver failure, Systematic review, Meta-analysis

## Abstract

**Background:**

Recent studies have shown that mesenchymal stem cell (MSC) therapy has potential therapeutic effects for patients with end-stage liver diseases. However, a consensus on the efficacy and safety of MSCs has not been reached.

**Methods:**

A systemic literature review was conducted by searching the Cochrane Library and PubMed databases for articles that evaluated the impact of MSC therapy on the outcomes among patients with end-stage liver disease. Various parameters, including pre- and post-treatment model of end-stage liver disease (MELD) score, serum albumin (ALB), total bilirubin (TB), coagulation function, aminotransferase, and survival rate, were evaluated.

**Results:**

This meta-analysis included a final total of 13 studies and 854 patients. The results indicated improved liver parameters following MSC therapy at different time points, including in terms of MELD score, TB level, and ALB level, compared with conventional treatment. Furthermore, the MSC treatment increased the overall survival rate among patients with liver cirrhosis and acute-on-chronic liver failure (ACLF). The changes in transaminase level and coagulation function differed between the different therapies at various post-treatment time points, indicating that MSC therapy provided no significant benefits in this regard. The further subgroup analysis stratified by liver background revealed that patients with ACLF benefit more from MSC therapy at most time points with improved liver function, including in terms of MELD score, TB level, and ALB level. In addition, no serious side effects or adverse events were reported following MSC therapy.

**Conclusions:**

The meta-analysis results suggest that MSC therapy is safe and results in improved liver function and survival rates among patients with end-stage liver disease. The subgroup analysis stratified by liver background indicated that patients with ACLF benefit more from MSC therapy than patients with liver cirrhosis at most time points.

**Supplementary Information:**

The online version contains supplementary material available at 10.1186/s13287-023-03494-2.

## Introduction

Mesenchymal stem cells (MSCs), as one of the multipotent cells, have the potential to self-renew and differentiate into multiple types of cells, such as epithelial cells or hepatocytes [1, 2]. Studies in animal models have shown that MSC therapy can improve liver function, ameliorate liver fibrosis and reverse acute hepatic failure [3–5]. Therefore, MSCs are believed to repair damaged hepatocytes and livers, providing therapeutic approaches for end-stage liver disease.

In the clinical setting, autologous and allogeneic MSC infusion is most often instituted in the treatment of liver cirrhosis and liver failure [6]. Compared with autologous MSC treatment, allogenic MSCs overcome the problems of long preparation and delays [7]. In addition, improved cell differentiation, proliferation, and cytokine secretion are provided by allogeneic MSCs derived from healthy donors [8]. In recent decades, studies have shown that MSC treatment can significantly improve liver function and ameliorate liver fibrosis in patients with decompensated liver cirrhosis [9]. Survival rates for patients with acute-on-chronic liver failure (ACLF) are also improved without increased side effects in the long-term [10].

Although a number of studies have been performed to evaluate the benefits of MSC treatment in end-stage liver disease, its clinical efficacy and safety remain unclear. Few meta-analyses of MSC therapy assessed treatment based on controlled trials or consistent evaluation variables. Additionally, detailed analyses of different end-stage liver diseases, including decompensated liver cirrhosis and acute-on-chronic liver failure, were not performed. Therefore, we conducted a meta-analysis of available comparative research to assess the clinical value and safety of MSCs in decompensated liver cirrhosis and ACLF.

## Materials and methods

### Literature search

Two independent investigators searched the PubMed and Cochrane Library databases (April 2022) to retrieve relevant studies. Comparative trials evaluating the therapeutic value and safety of MSCs versus a control in the treatment of decompensated liver cirrhosis and ACLF were included. No restrictions were set for language, publication date, or publication status. The search strategy was based on the following keywords: “mesenchymal stem cells” and “liver cirrhosis” or “decompensated liver cirrhosis” or “liver failure” or “acute-on-chronic liver failure” and “clinical study” or “clinical trial” or “randomized controlled trial” or “randomized clinical trial”.

### Inclusion and exclusion criteria

The main inclusion criteria were comparative studies evaluating outcomes between MSC therapy versus a control in the treatment of decompensated liver cirrhosis and ACLF. The exclusion criteria were as follows: (1) non-comparative studies, case reports, letters, reviews, editorials; (2) studies that lacked clinical data or outcomes; (3) if multiple studies were reported by the same institution, only the highest quality study was included.

### Data extraction

Two independent investigators reviewed texts, figures, and tables to extract information from the included studies. The following data were collected: (1) first author name, year of publication, country and study type; (2) study sample size; (3) cell type, cell dosage of MSCs, and time of treatment; (4) study outcomes including albumin (ALB), total bilirubin (TB), model for end-stage liver disease (MELD) score, coagulation function, liver transaminase level, adverse events, and survival rates.

### Quality assessment

The Newcastle–Ottawa scale (NOS) was used to assess the quality of included studies [11].

### Statistical analysis

The dichotomous variables were evaluated using odds ratios (ORs) with a 95% confidence interval (CI). In the survival analysis, the OR indicated the relative likelihood of death in each group. Continuous variables were assessed by weighted mean differences (WMD). Higgins I^2^ statistic was used to assess statistical heterogeneity among studies. When I^2^ < 50%, a fixed-effects model was used for calculations. On the other hand, when heterogeneity was greater than 50%, a random-effects model was recommended. Funnel plots, Egger’s and Begg’s tests were used to identify publication bias. This meta-analysis was performed using Review Manager version 5.3 (Revman, The Cochrane Collaboration, Oxford, UK). *P* < 0.05 was considered statistically significant.

## Results

### Study selection and eligibility

**Fig. 1:**
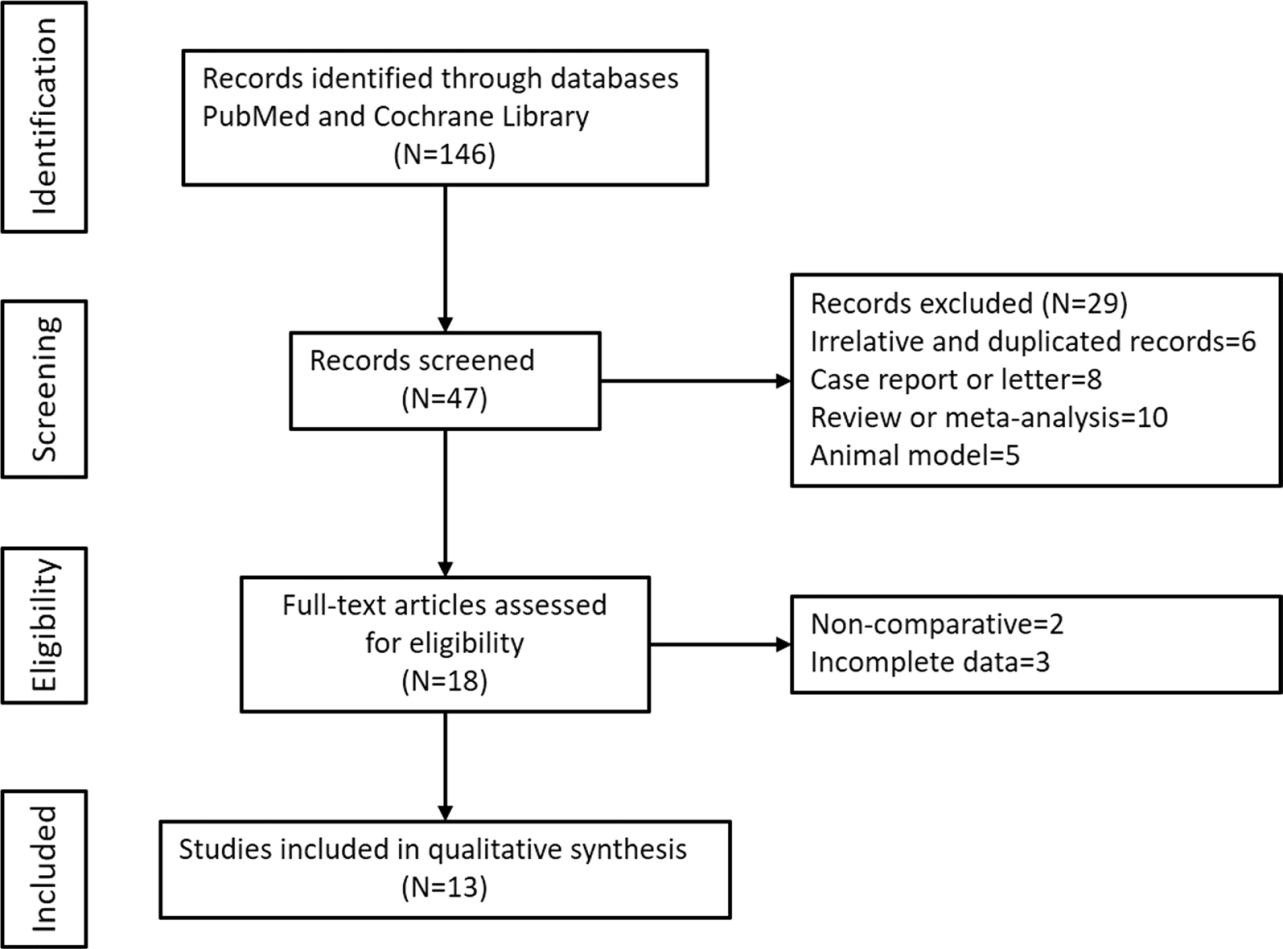
Flow diagram of article and study selection process

The search strategy initially generated 47 studies, as shown in the flowchart (Fig. 1). Twenty-nine of these studies were excluded because of lack of relevance, duplication, or review article type. Another five studies did not meet the inclusion criteria due to insufficient data or improper study type. Finally, a total of 13 studies were enrolled in our study [12–24].

A total of 854 patients from five countries were included in the meta-analysis. The patient demographics and baseline characteristics of the included studies are given in Table 1. The studies included patients with end-stage liver diseases including decompensated liver cirrhosis (n = 7) and ACLF (n = 6), of which eight were conducted in China, two in Egypt, one in Korea, one in Brazil, and one in Iran. Four hundred and three patients received MSC therapy, and 451 patients in the control group underwent supportive therapy. MSCs were divided into two types: originating from the umbilical cord (UC-MSCs, n = 5) and from the bone marrow (BM-MSCs, n = 8).

**Table 1:** Clinicopathological characteristics of the included studies

Year	Author	Country	Liver disease	Cell type	Cell dosage	Administration route	Patient number		Median age		Follow-up duration
							Exp group	Con group	Exp group	Con group	
2021	Shi	China	Decompensated cirrhosis	Allogeneic UC-MSCs	1 × 10^6^/kg	Intravenous injection	108	111	47	48	75 months
2021	Schacher	Brazil	ACLF	Allogeneic BM-MSCs	1 × 10^6^/kg	Intravenous injection	4	5	55.8	53.4	90 days
2019	Xu	China	ACLF	Allogeneic UC-MSCs	1 × 10^5^/kg	Intravenous injection	30	30	40.7	44.7	48 weeks
2017	Lin	China	ACLF	Allogeneic BM-MSCs	(1–10) × 10^5^/kg	Intravenous injection	56	54	40	42.8	24 weeks
2016	Suk	South Korea	Decompensated cirrhosis	Autologous BM-MSCs	5 × 10^7^/kg	Hepatic arterial injection	18	18	53.1	53.7	12 months
2016	Li	China	ACLF	Allogeneic UC-MSCs	100 × 10^6^/kg	Hepatic arterial injection	11	34	51.1	50.0	24 months
2014	Xu	China	Decompensated cirrhosis	Autologous BM-MSCs	0.75 ± 0.5 × 10^6^	Hepatic arterial injection	20	19	44	45	24 weeks
2014	Salama	Egypt	Decompensated cirrhosis	Autologous BM-MSCs	1 × 106/kg	Intravenous injection	20	20	50.3	50.9	6 months
2013	Mohamadnejad	Iran	Decompensated cirrhosis	Autologous BM-MSCs	(1.2–2.95) × 10^8^/kg	Intravenous injection	14	11	43.1	34.6	12 months
2012	Zhang	China	Decompensated cirrhosis	Allogeneic UC-MSCs	0.5 × 10^6^/kg	Intravenous injection	30	15	48	47	48 weeks
2012	Shi	China	ACLF	Allogeneic UC-MSCs	0.5 × 10^7^/kg	Intravenous injection	24	19	40	45	48 weeks
2012	EI-Ansary	Egypt	Decompensated cirrhosis	Autologous BM-MSCs	1 × 10^6^/kg	Intravenous injection	15	10	48	51.6	6 months
2011	Liang	China	ACLF	Autologous BM-MSCs	(3.4 ± 3.8) × 10^8^	Hepatic arterial injection	53	105	42.2	42.2	192 weeks

### Quality assessment

Newcastle–Ottawa scale (NOS) was used to assess the quality of included studies. Four studies had NOS scores of nine, indicating high quality. The other nine studies were considered to be of moderate quality.

### Meta-analysis results

#### Survival rate

**Fig. 2:**
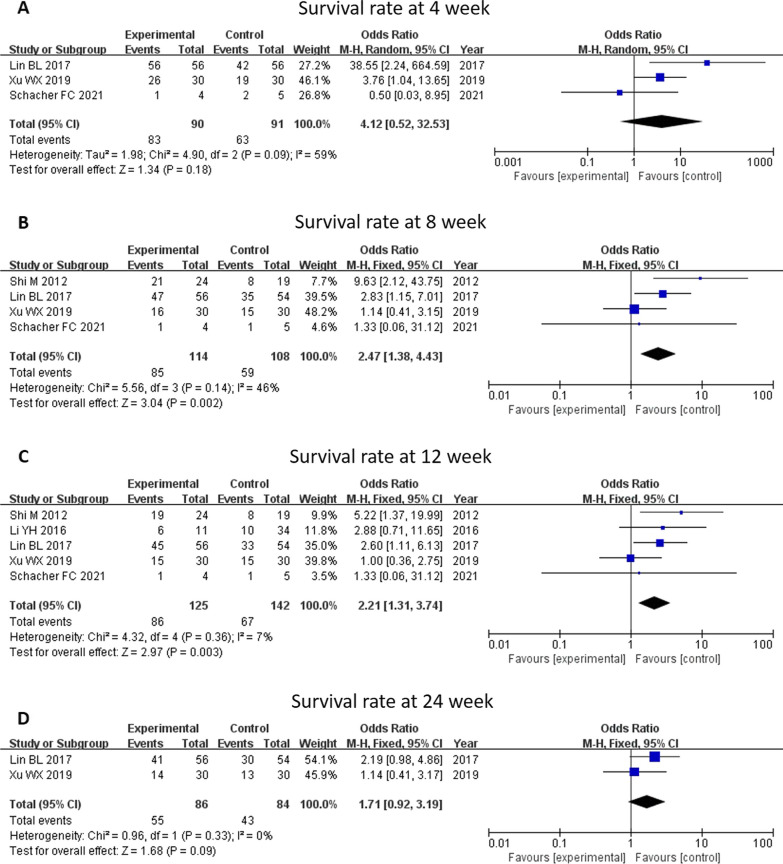
Meta-analysis of the comparison between mesenchymal stem cell (MSC) therapy and conventional treatment in terms of survival rate. **B** and **C** The patients receiving MSC therapy had a higher chance of survival at 8 and 12 weeks compared with the controls. **A** and **D** The MSC therapy did not have a significant survival benefit at 4 or 24 weeks

Survival rates of patients at 4 weeks, 8 weeks, 12 weeks, and 24 weeks were analyzed (Fig. 2). Patients receiving MSC therapy had a higher chance of survival at 8 (OR 2.47, 95% CI 1.38–4.43, *P* = 0.002) and 12 weeks (OR 2.21, 95% CI 1.31–3.74, *P* = 0.003) compared with controls. While MSC therapy did not show a significant survival benefit at 4 weeks (OR 4.12, 95% CI 0.52–32.53 *P* = 0.18) or 24 weeks (OR 1.71, 95% CI 0.92–3.19, *P* = 0.09), it tended to increase survival rate at these points. Subgroup analysis of survival at 8 weeks and 12 weeks by different liver disease backgrounds was performed. All included patients were diagnosed as ACLF before treatment; thus, MSC therapy was associated with increased survival rate at 8 weeks and 12 weeks in the ACLF group.

#### MELD score

**Fig. 3:**
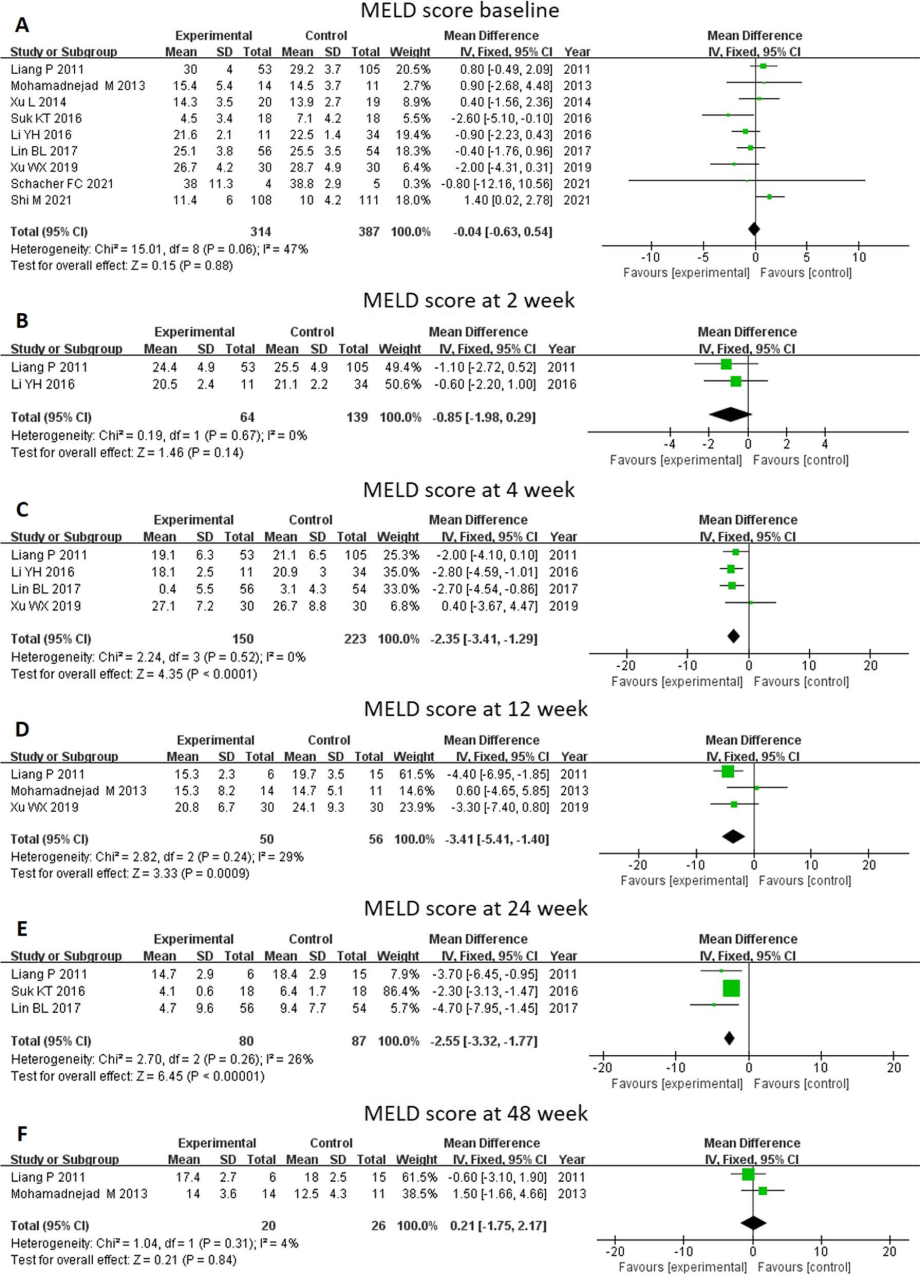
Meta-analysis of the comparison between MSC therapy and conventional treatment in terms of model of end-stage liver disease (MELD) score. **A** There was no difference in baseline MELD score between the MSC group and the control group. **C**, **D** and **E** The MELD score decreased significantly at 4, 12, and 24 weeks following MSC therapy. **B** and **F** No significant difference was found between the two therapy types at 2 and 48 weeks

**Fig. 4:**
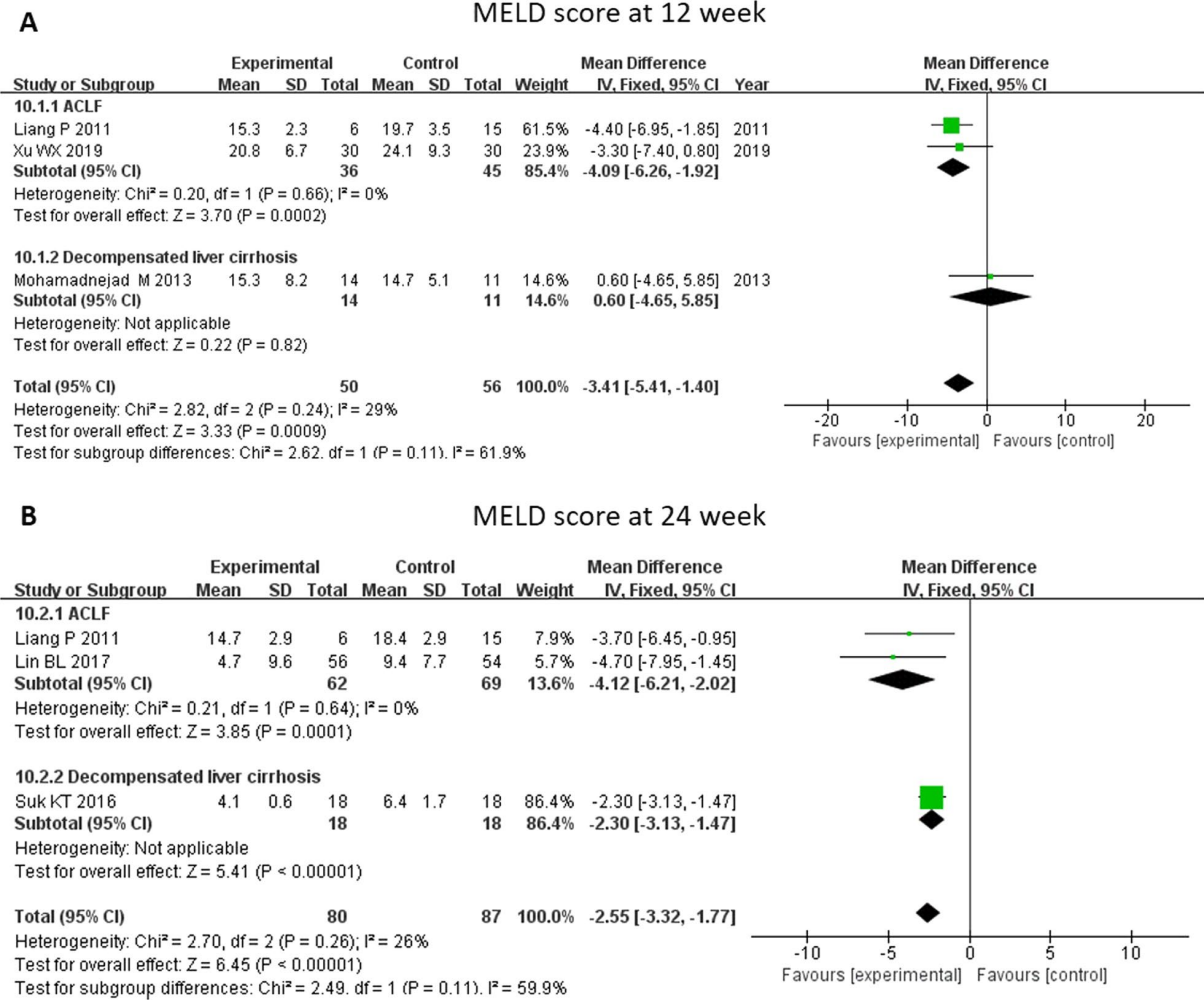
Meta-analysis of the comparison between MSC therapy and conventional treatment in terms of MELD score, as stratified by different liver diseases. **A** and **B** The patients with acute-on-chronic liver failure (ACLF) had a significantly decreased MELD score following MSC therapy at 12 and 24 weeks. **B** A decreased MELD score at 24 weeks was observed among the patients with cirrhosis following MSC therapy

The MELD score was calculated according to a formula using three laboratory test results for bilirubin, prothrombin time, and creatinine. Nine studies included an analysis of the MELD score to enable a rapid evaluation of the urgent need of a liver transplantation among the candidates (Fig. 3). Baseline MELD score was not different between the MSC and control groups (MD -0.04, 95% CI -0.63–0.54, p = 0.88). The MELD score decreased significantly at 4 weeks (MD -2.35, 95% CI -3.41- -1.29, *P* < 0.0001), 12 weeks (MD -3.41, 95% CI -5.41- -1.40, *P* = 0.0009) and 24 weeks (MD -2.55, 95%CI -3.32- -1.77, *P* < 0.0001) through MSC therapy. No significant difference was found between the two therapies at 48 weeks. Further subgroup analysis showed that patients with ACLF had a significantly decreased MELD score by MSC therapy at 4 weeks, 12 weeks (MD -4.09, 95% CI -6.26- -1.92, *P* = 0.0002), and 24 weeks (MD -4.12, 95% CI -6.21- -2.02, *P* = 0.0001). For patients with cirrhosis, a decreased MELD score at 24 weeks was observed after MSC therapy (MD -2.30, 95% CI -3.13- -1.47, *P* < 0.00001) (Fig. 4).

#### ALB level

**Fig. 5:**
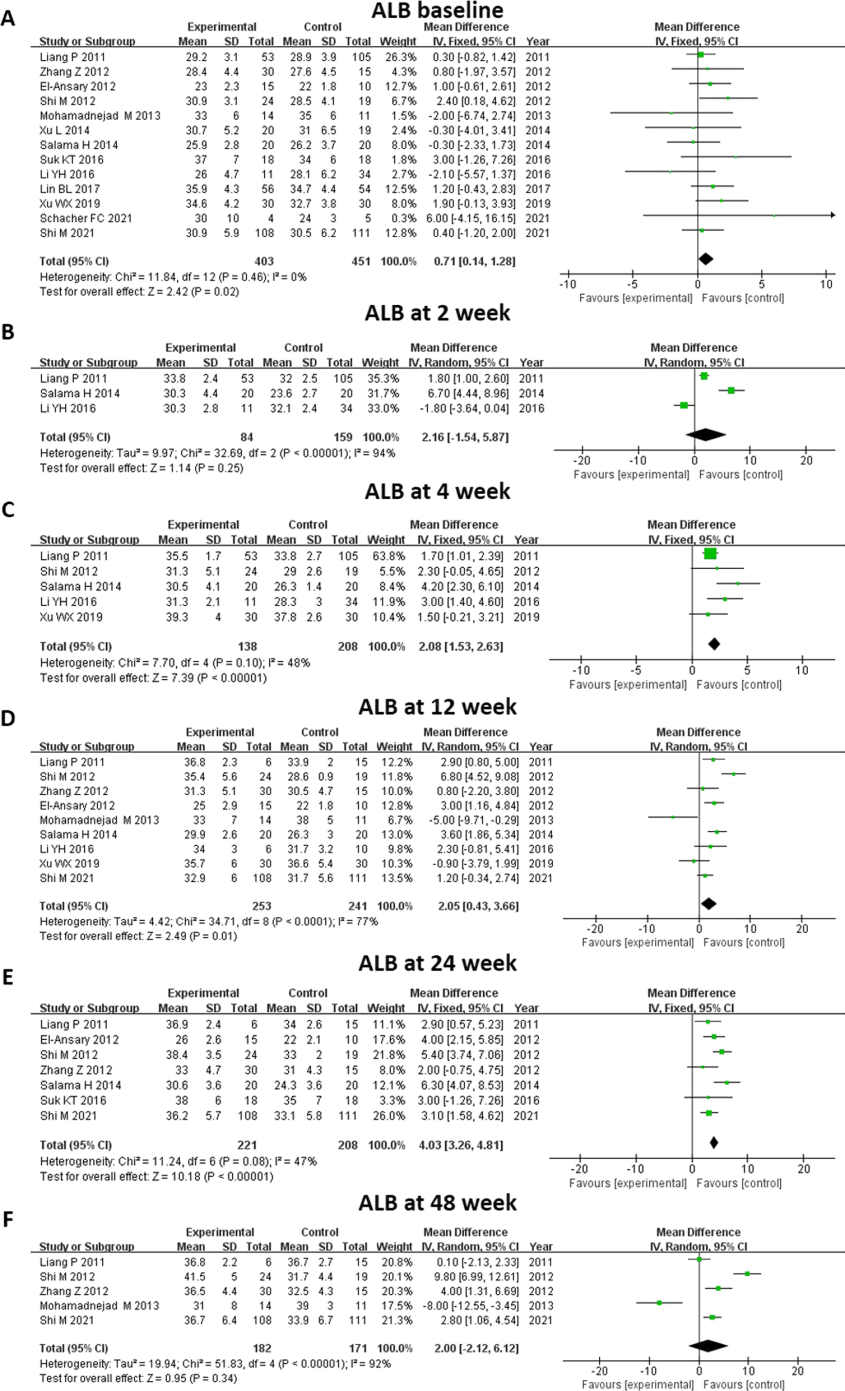
Meta-analysis of the comparison between MSC therapy and conventional treatment in terms of albumin (ALB) level. **A**, **B** and **F** There was no significant difference between the MSC group and the control group at baseline and at 2 and 48 weeks. **C**, **D** and **E** Compared with the controls, the ALB level was significantly elevated at 4, 12 and 24 weeks among the patients who received MSC therapy

**Fig. 6:**
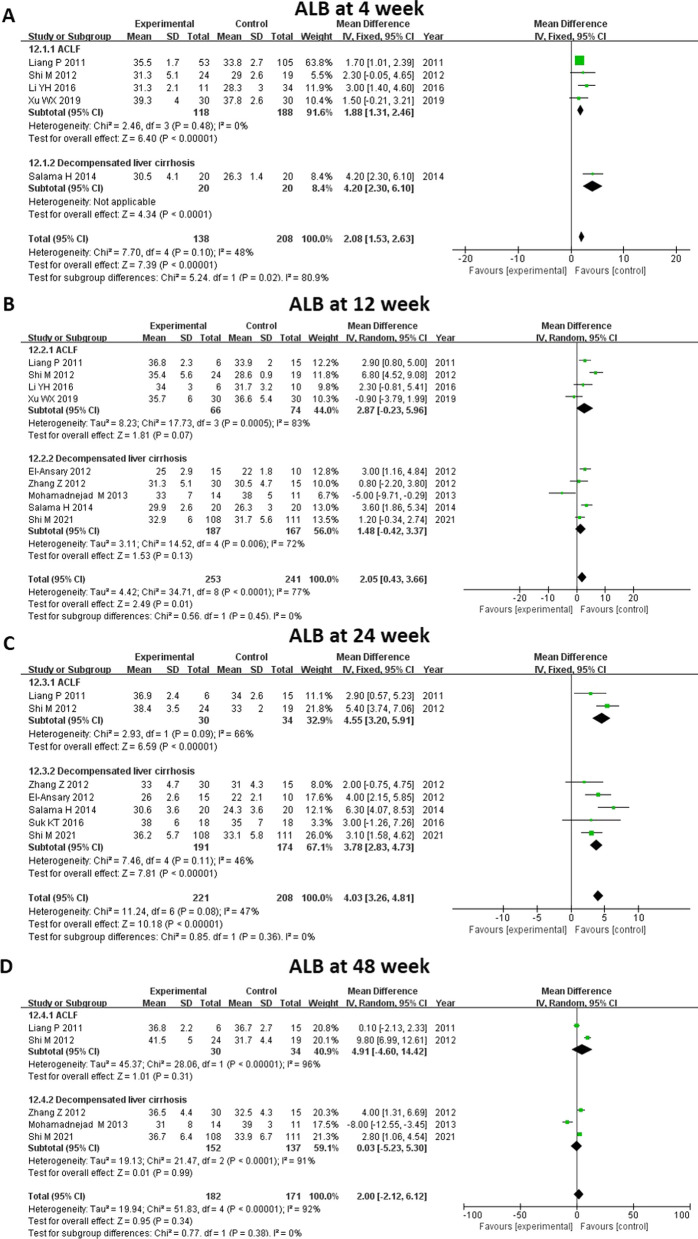
Meta-analysis of the comparison between MSC therapy and conventional treatment in terms of ALB level, as stratified by different liver diseases. **A** and **C** The MSC therapy was related to an increased level of ALB at 4 and 24 weeks in both the ACLF subgroup and the cirrhosis subgroup. **B** and **D** No benefits of MSC therapy were observed at all other time points

All thirteen studies were enrolled in the analysis of ALB level (Fig. 5). Little difference was seen between the MSC and control groups at baseline (MD 0.71, 95% CI 0.14–1.28, *P* = 0.02). Compared with controls, ALB was significantly elevated in those who received MSC therapy at 4 weeks (MD 2.08, 95% CI 1.53–2.63, *P* < 0.00001), 12 weeks (MD 2.05, 95% CI 0.43–3.66, *P* = 0.01), and 24 weeks (MD 4.03, 95% CI 3.26–4.81, *P* < 0.00001). We did further subgroup analysis to explore whether liver disease background influenced ALB level after treatment, which showed that MSC therapy was related to an increased level of ALB at 4 weeks (MD 1.88, 95% CI 1.31–2.46, *P* < 0.00001) and 24 weeks (MD 4.55, 95% CI 3.20–5.91, *P* < 0.0001) in both the ACLF and cirrhosis subgroups (Fig. 6).

#### TB level

**Fig. 7:**
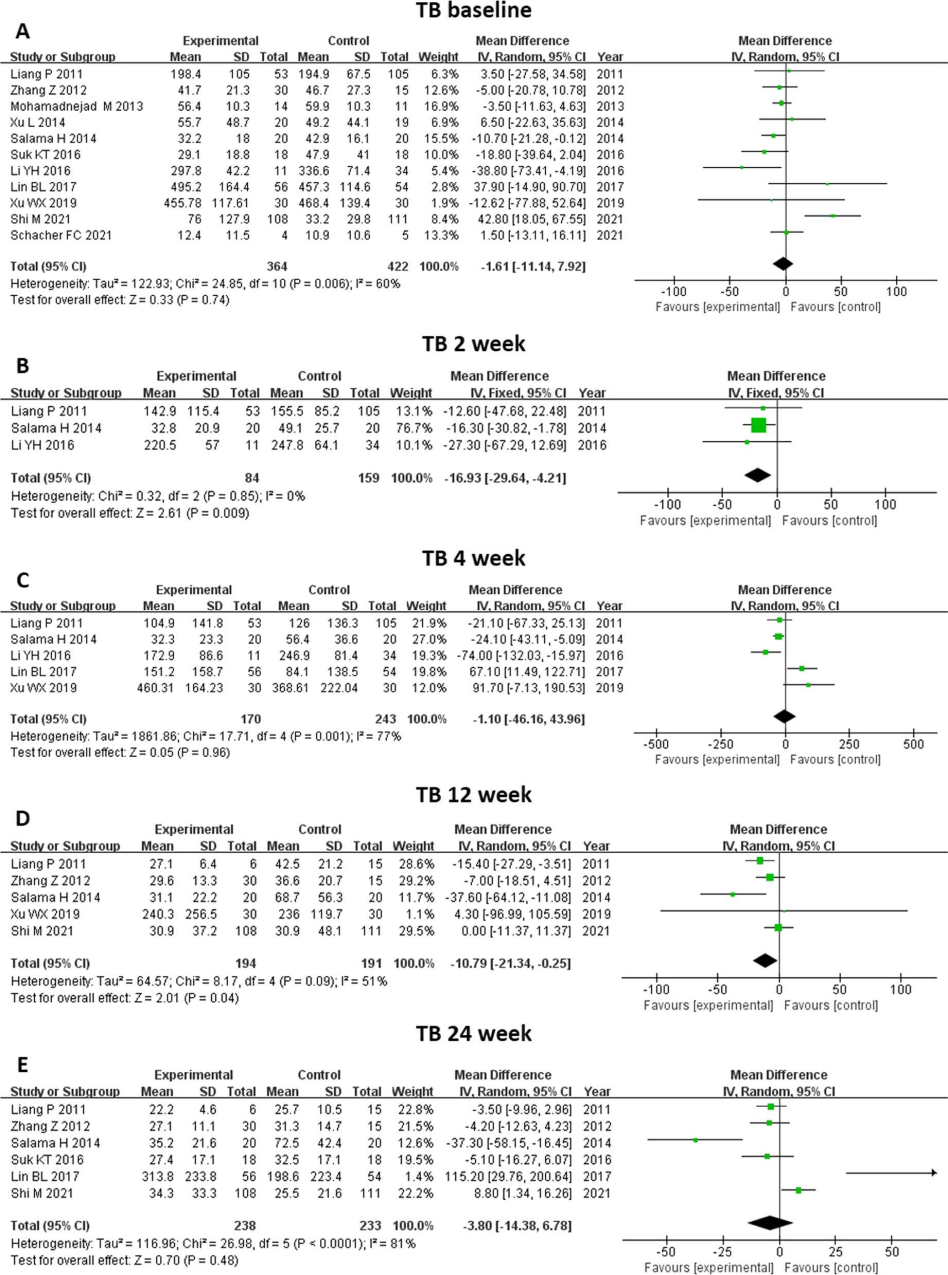
Meta-analysis of the comparison between MSC therapy and conventional treatment in terms of total bilirubin (TB) level. **A** The TB level before treatment was not significantly different between the two groups. **B** and **D** The TB level decreased significantly at 2 and 12 weeks following MSC therapy. **C** and **E** No significant changes were observed following the two treatments at 4 and 24 weeks

**Fig. 8:**
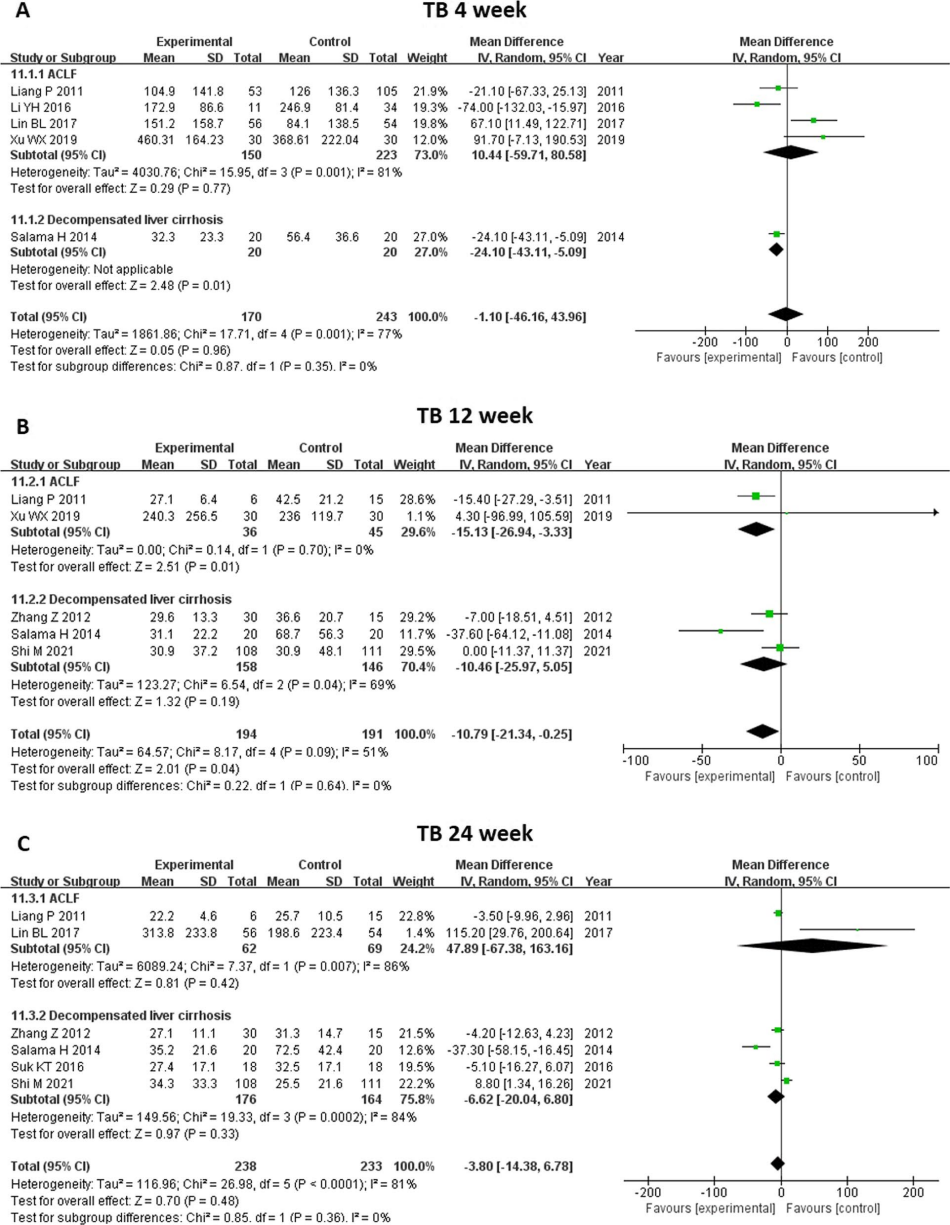
Meta-analysis of the comparison between MSC therapy and conventional treatment in terms of total bilirubin (TB) level, as stratified by different liver diseases. **A** The MSC therapy led to a reduction in TB level at 4 weeks among the patients with cirrhosis and **B** at 12 weeks among the patients with ACLF. **C** No benefit of MSC therapy in terms of TB level was observed at 24 weeks

We included eleven studies in the analysis of TB level (Fig. 7). TB level before treatment was not significantly different between the two groups (MD -1.61, 95% CI -11.14–7.92, *P* = 0.74). TB level decreased significantly at 2 weeks (MD -16.93, 95%CI -29.64- -4.21, p = 0.009) and at 12 weeks (MD -10.79, 95%CI -21.34- -0.25, p = 0.04) after MSC therapy. No significant changes were found after two treatments at 4 weeks and at 24 weeks. Further subgroup analysis showed that MSC therapy led to a reduction in TB level at 12 weeks in patients with ACLF (MD -15.13, 95% CI -26.94- -3.33, *P* = 0.01), and at 4 weeks in patients with cirrhosis (MD -24.10, 95% CI -43.11- -5.09, *P* = 0.01) (Fig. 8).

#### Coagulation function

**Fig. 9:**
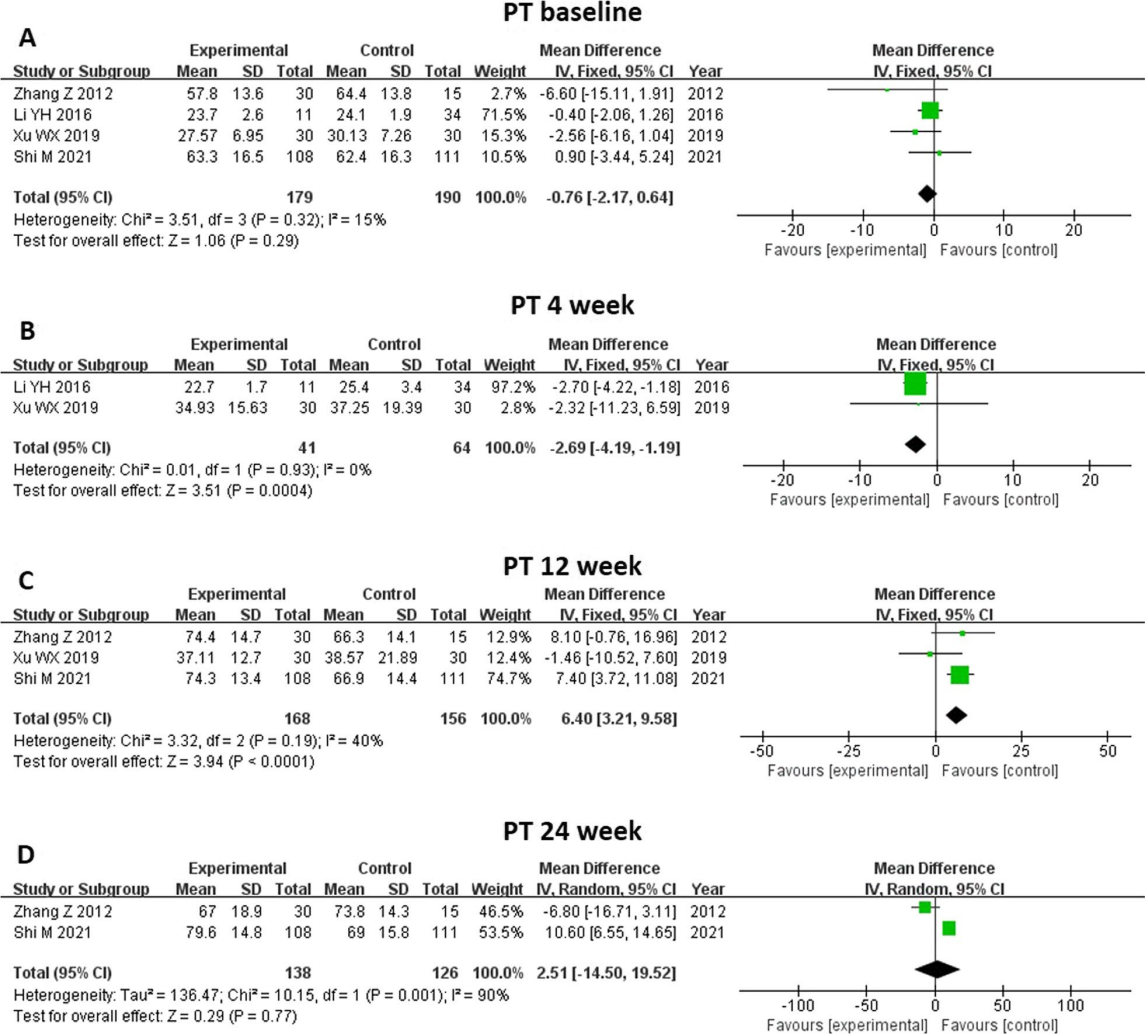
Meta-analysis of the comparison between MSC therapy and conventional treatment in terms of prothrombin activity (PT). **A** There was no difference in baseline PT level between the two groups. **B** Compared with the control groups, the MSC treatment significantly decreased the participants’ PT level at 4 weeks. **C** The PT level increased at 12 weeks following MSC treatment and **D** did not differ between the groups at 24 weeks

**Fig. 10:**
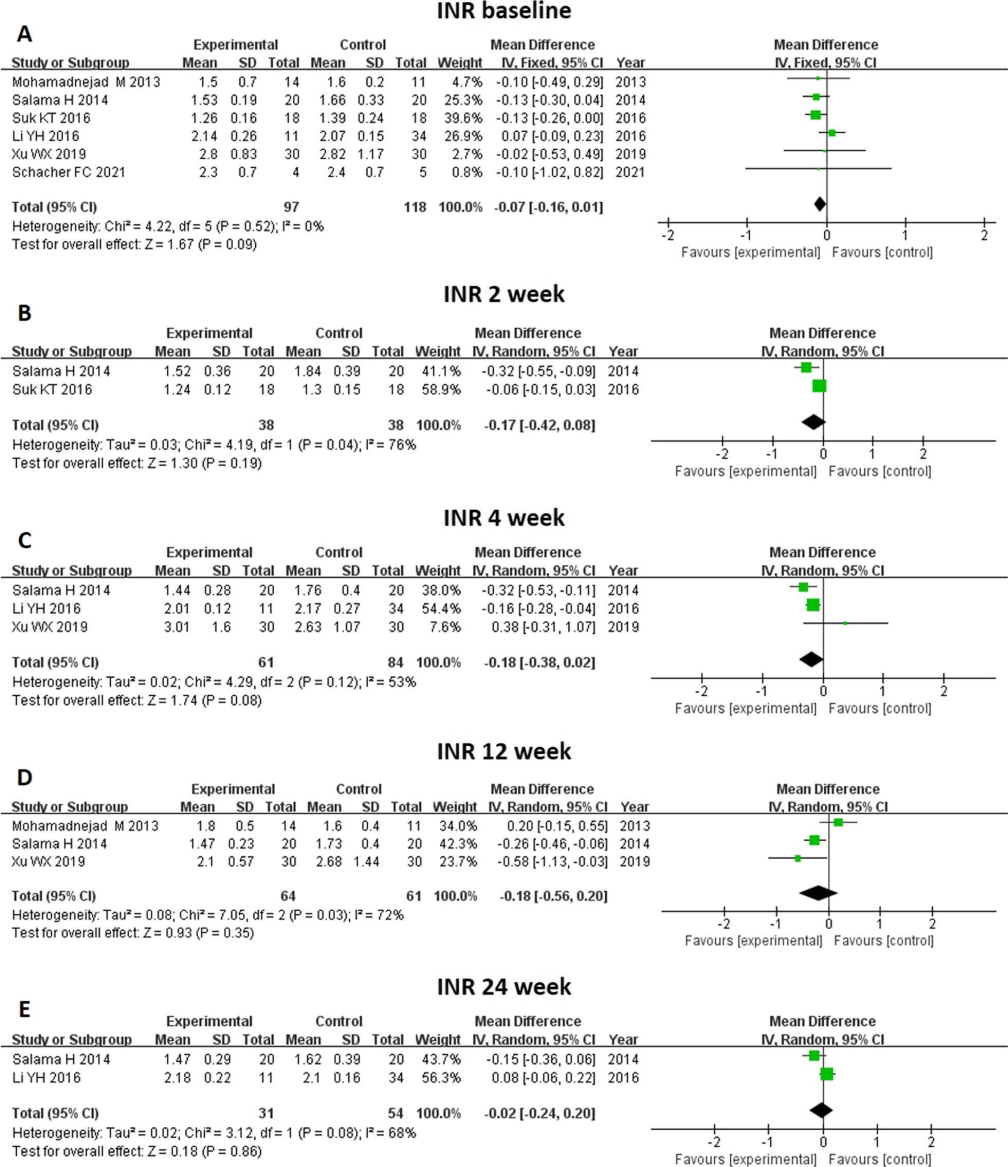
Meta-analysis of the comparison between MSC therapy and conventional treatment in terms of international normalized ratio (INR). No significant changes were observed before and after both treatments at all time points. **A** There was no difference in baseline INR level between the two groups. **B**, **C**, **D** and **E** The INR level did not differ between the groups at 2, 4, 12 and 24 weeks

Four studies and six studies reported changes in prothrombin time (PT) and international normalized ratio (INR) at different time points, respectively (Figs. 9 and 10). Baseline PT level was not different between the two groups (MD -2.17, 95% CI -2.17–0.64, *P* = 0.29). Compared with control groups, MSC treatment decreased participants’ PT level significantly at 4 weeks (MD -2.69, 95% CI -4.19–1.19, *P* = 0.0004). However, PT level increased at 12 weeks after MSCs treatment (MD 6.40, 95% CI 3.21–9.58, *P* < 0.0001) and did not differ between the groups at 24 weeks (MD 2.51, 95% CI -14.50–19.52, *P* = 0.77). For INR level, no significant changes were found before and after both treatments at other time points. Due to limited included studies, subgroup analysis was not performed.

#### Transaminase level (ALT and AST)

**Fig. 11:**
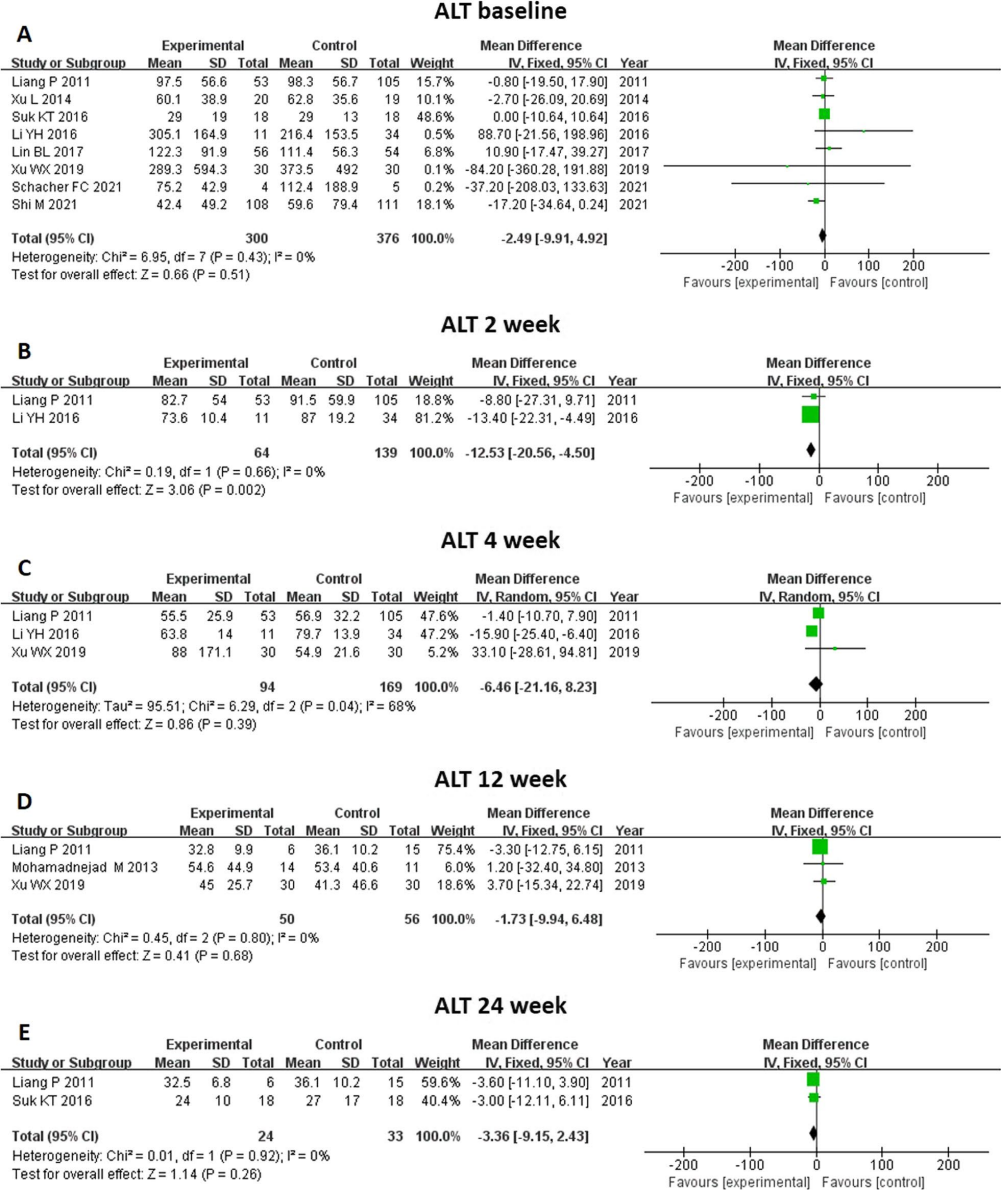
Meta-analysis of the comparison between MSC therapy and conventional treatment in terms of alanine aminotransferase (ALT) level. **A** There was no significant difference in baseline ALT level between the two groups. **B** Following MSC treatment, the ALT level at 2 weeks decreased significantly compared with the control group (**B**). **C**, **D** and **E** Following MSC treatment, there were no significant changes in ALT level at 4, 12 and 24 weeks

**Fig. 12:**
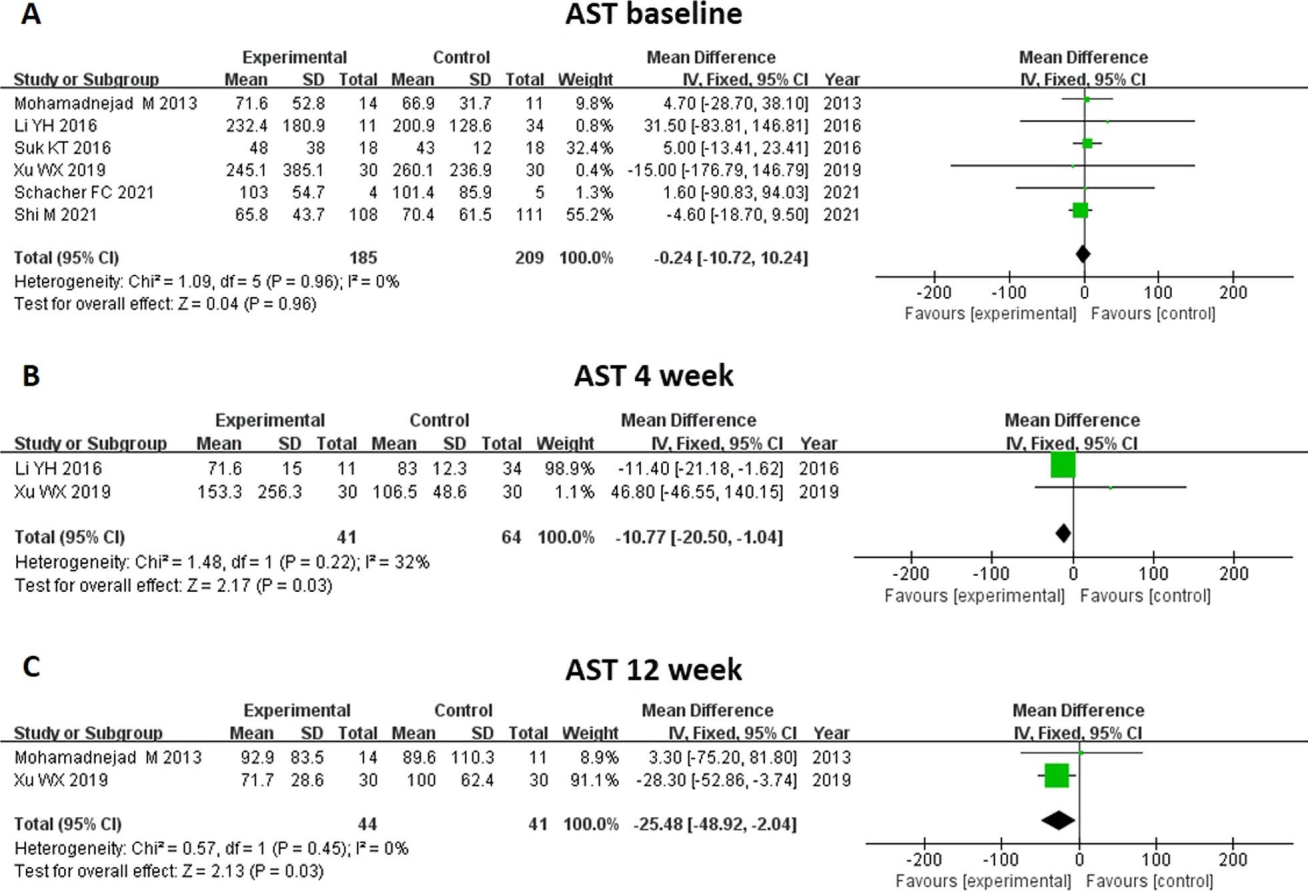
Meta-analysis of the comparison between MSC therapy and conventional treatment in terms of aspartate aminotransferase (AST) level. **A** There was no significant difference in baseline AST level between the two groups. **B** and **C** Following MSC therapy, the AST level was significantly decreased at 4 and 12 weeks

An analysis of ALT and AST levels was reported by eight and five studies, respectively (Figs. 11 and 12). There was no significant difference in baseline transaminase level between groups. Through MSC treatment, the ALT level at 2 weeks (MD − 12.53, 95% CI − 20.56 to − 4.5, *P* = 0.002) decreased significantly compared to control group. However, ALT level after MSC treatment at 4, 12, and 24 weeks did not show significant changes. As for AST level, MSC therapy significantly decreased the AST level at 4 weeks (MD − 10.77, 95% CI − 20.50 to − 1.04, *P* = 0.03) and 12 weeks (MD -25.48, 95%CI -48.92- -2.04, p = 0.03).

#### Adverse events and complications

No significant adverse events or complications related to MSC therapy were reported by seven studies. Six studies reported major complications, including encephalopathy, gastrointestinal hemorrhage, rash, and infection. Analysis showed that encephalopathy was significantly reduced after MSC therapy (OR 0.41, 95% CI 0.18–0.91 *P* = 0.03), while the clinical symptoms of gastrointestinal hemorrhage, rash, and infection did not differ between MSC therapy and control groups. There was no statistically significant difference in long-term risk of development of hepatocellular carcinoma between the two groups (OR 0.58, 95% CI 0.29–1.15 *P* = 0.12) (Table 2).

**Table 2:** Meta-analysis of major complications after therapy

Major complications	Number of studies	Heterogeneity (I^2^) (%)	Odds ratio (OR)	95%CI	*P* value
Encephalopathy	3	0	0.41	0.18–0.91	0.03
Gastrointestinal hemorrhage	5	0	0.70	0.26–1.88	0.48
Rash	2	0	1.3	0.34–5.01	0.69
Infection	2	64	0.79	0.16–3.99	0.78
HCC development	3	0	0.58	0.29–1.15	0.12

## Discussion

In recent years, studies have demonstrated that MSC therapy is a safe and effective treatment for chronic liver diseases [25, 26]. This systematic review and meta-analysis aimed to evaluate the safety and efficacy of MSC treatment for decompensated liver cirrhosis and ACLF. Our results showed that MSC therapy might improve liver function, assessed by MELD score, TB, and ALB levels. Additionally, MSC treatment appeared to improve survival in patients. No significant difference in transaminase levels or coagulation function was observed between MSC and conventional treatment.

In the present study, the first controversial issue is the effect of MSC treatment on TB and transaminase [27]. Though MSC treatment could improve liver function compared with the baseline, pooled results suggested that MSC treatment could not markedly improve TB and transaminase at all time points. In 2021, Schacher et al. [18] pointed out that end-stage liver disease that results in persistent liver injury may be the reason for the discrepancy. The different liver diseases and the limited sample sizes may explain why statistically significant differences in ALT levels were not detected between the groups, which was consistent with studies conducted in 2017 and 2021 [15, 16, 18].

Another important point relates to the impact of MSC treatment on the survival of patients. For patients with ACLF, it is important to determine whether patients can survive for the first 3 months, since the mortality of whom can be as high as 65%. In our analysis, all included patients were diagnosed as ACLF before treatment and they had a higher chance of survival at 4, 8 and 12 weeks after treatment with MSC. Besides, there was also a trend toward higher survival rates at 24 weeks treated with MSC. The results showed that MSC treatment could help ACLF patients survive for the first 3 months and even longer. It is noteworthy that long-term survival (beyond 48 weeks) was infrequently reported by the included studies. Recently in 2021, Shi et al. [20] reported a survival benefit in decompensated liver cirrhosis patients with MSC treatment over a 75-month follow-up, indicating that MSC therapy could improve the long-term outcomes in liver cirrhosis. Future long-term studies are required to confirm the survival benefits of MSC treatment in end-stage liver disease.

To further explore whether liver disease background influences the efficacy of MSC therapy, subgroup analysis stratified by decompensated liver cirrhosis and ACLF was performed. Owing to the small number of eligible studies, subgroup analysis was limited to TB, ALB, MELD score, and survival rate. It turns out that patients with ACLF might benefit more from MSC therapy at most time points. The results were in line with several studies and highlighted the regenerative role of MSCs in ACLF in 2021[14, 19, 28]. Previously, liver transplantation was regarded as the only therapeutic alternative in end-stage liver disease, especially ACLF [29]. The present study showed that MSC therapy may provide another potential choice in the treatment of ACLF.

Apart from the efficacy of MSC therapy in end-stage liver disease, different routes of MSC transfusion, which can either be through the peripheral vein or through the hepatic artery, are another concern in clinical practice. Theoretically, hepatic arterial injection is more effective than the peripheral vein route due to less loss of MSCs and the higher homing ability. However, the disadvantages of the invasive procedure and the higher risk of bleeding through hepatic arterial injection have been reported in many studies. Meanwhile, peripheral intravenous infusion is considered an ideal route as it is convenient to perform and the MSCs migrate well into the liver parenchyma and differentiate into hepatocytes in vivo. In our analysis, no significant difference was observed between the two routes (Additional file 1: Table S1–S3). More clinical studies are required to determine both the effectiveness and the convenience of the two different transfusion routes.

In addition, we also compared different cell types of MSCs on efficacy for end-stage liver disease. The results showed that BM-MSCs and UC-MSCs had little difference on improvement of liver function. However, previous studies suggested that UC-MSCs had better efficacy, since UC-MSCs showed low alloreactivity and young cellular age. Therefore, comparison of therapeutic effects between BM-MSCs and UC-MSCs calls for more clinical trials. It is noted that MSCs were originally named to represent a class of cells from human bone marrow and periosteum that could maintain their in vitro capacity to be induced to hepatocytes and tissues. Several reports in the early 2000s have described MSC-into-Hep maturation, which impelled clinical studies to confirm the beneficial effects of MSCs. However, recently, Dr. Caplan recommended to change the name of MSCs to Medicinal Signaling Cells since the assumption that MSCs differentiate into mature and functional hepatocytes has never been totally described or approved [30]. Instead, the paracrine action of the multipotent cells rather than differentiation capacity is believed to lead to regeneration induction. The controversy motivates more experimental and clinical studies to explore the differentiation capacity of these cells in end-stage liver disease.

Our meta-analysis has some limitations. First, only 13 reports were included in our study. Second, subgroup analysis of coagulation function and transaminase levels was missing owing to limited included studies. Also, subgroup analysis stratified by different cell types, times of treatment, and administration routes was not conducted, which may cause selection bias. Moreover, the sample size of most included studies was relatively small and long-term follow-up was lacking. Future multi-center large-scale studies are required to further evaluate the efficacy of MSC treatment. Finally, most of the included studies were performed by countries in Asia. This mainly contributes to high incidence of viral hepatitis and liver cirrhosis in Asian countries. This factor is perhaps another potential source of bias.

## Conclusions

Despite the limitations noted above, our results incorporated the data from 854 patients to evaluate the safety and efficacy of MSC therapy in the treatment of end-stage liver disease. The results indicated that MSC therapy improved the liver function at most time points, including in terms of MELD score, TB level, and ALB level, compared with conventional treatment. Furthermore, the MSC treatment increased the overall survival rate among the patients. The further subgroup analysis stratified according to liver background revealed that patients with ACLF benefit more from MSC therapy at most time points, with improved liver function. However, there remain concerns regarding MSC source, administration route, and long-term outcomes. Therefore, future multi-center large-scale studies are required to confirm the efficacy and safety of MSC treatment in decompensated liver cirrhosis and ACLF.

### Supplementary Information

**Additional file 1**: **Table S1** Results of administration route and cell type of MSCs therapy on MELD score. **Table S2** Results of administration route and cell type of MSCs therapy on ALB level. **Table S3** Results of administration route and cell type of MSCs therapy on TBIL level..

## Data Availability

The data are available from the corresponding authors upon reasonable request.

## Abbreviations

MSC: Mesenchymal stem cells
ACLF: Acute-on-chronic liver failure
MELD: End-stage liver disease
ALB: Serum albumin
TB: Total bilirubin
OR: Odds ratios
CI: Confidence interval
WMD: Weighted mean differences
PT: Prothrombin time
INR: International normalized ratio
ALT: Alanine aminotransferase level
AST: Aspartate aminotransferase level
