# Supplementary material for: Meta-analysis on last ten years of clinical injection of bone marrow-derived and umbilical cord MSC to reverse cirrhosis or rescue patients with acute-on-chronic liver failure
Source: Stem Cell Res Ther. 2023 Sep 23;14:267. doi: 10.1186/s13287-023-03494-2 (PMC10518116; doi:10.1186/s13287-023-03494-2)
Supplement: Supplementary file 1 — Additional file 1: Table S1 Results of administration route and cell type of MSCs therapy on MELD score. Table S2 Results of administration route and cell type of MSCs therapy on ALB level. Table S3 Results of administration route and cell type of MSCs therapy on TBIL level.. [file 13287_2023_3494_MOESM1_ESM.docx]

**Supplementary Table 1.** Results of administration route and cell type of MSCs therapy on MELD score

|  | **Time point** | **Heterogeneity** | | **SMD** | **95%-CI** | ***Z*** | ***p*-value** |
| --- | --- | --- | --- | --- | --- | --- | --- |
|  |  | ***I2*** | ***p-value*** |  |  |  |  |
| **Administration route** |  |  |  |  |  |  |  |
| Intravenous injection | Baseline | 42.8% | 0.136 | 0.06 | [-0.13, 0.25] | 0.61 | 0.544 |
|  | 4 week | 70.5% | 0.065 | -0.33 | [-0.63, -0.33] | 2.13 | 0.033 |
|  | 12 week | 5.0% | 0.305 | -0.26 | [-0.69, 0.17] | 1.19 | 0.232 |
|  | Overall | 54.4% | 0.012 | -0.14 | [-0.28, -0.01] | 2.09 | 0.037 |
| Hepatic arterial injection | Baseline | 67.1% | 0.028 | -0.04 | [-0.29,0.20] | 0.35 | 0.723 |
|  | 24 week | 0.0% | 0.556 | -1.46 | [-1.93,-0.99] | 6.11 | 0.000 |
|  | Overall | 76.1% | 0.000 | -0.34 | [-0.49,-0.18] | 4.22 | 0.000 |
| **Cell type** |  |  |  |  |  |  |  |
| BM-MSC | Baseline | 37.4% | 0.143 | -0.09 | [-0.33, 0.15] | 0.49 | 0.623 |
|  | 4 week | 0.0% | 0.362 | 0.49 | [-0.37, 1.36] | 3.23 | 0.001 |
|  | 12 week | 78.8% | 0.030 | 0.55 | [-1.38, 1.48] | 1.38 | 0.167 |
|  | 24 week | 70.3% | 0.018 | -0.39 | [-1.63, 0.86] | 6.01 | 0.000 |
|  | 48 week | 0.0% | 0.329 | 0.13 | [-0.48,0.74] | 0.41 | 0.683 |
|  | Overall | 0.670 | 0.000 | 0.01 | [-0.29, 0.31] | 5.03 | 0.000 |

**Supplementary Table 2.** Results of administration route and cell type of MSCs therapy on ALB level.

|  | **Time point** | **Heterogeneity** | | **SMD** | **95%-CI** | ***Z*** | ***p*-value** |
| --- | --- | --- | --- | --- | --- | --- | --- |
|  |  | ***I2*** | ***p-value*** |  |  |  |  |
| **Administration route** |  |  |  |  |  |  |  |
| Intravenous injection | Baseline | 11.5% | 0.341 | 0.20 | [0.01, 0.39] | 2.07 | 0.039 |
|  | 4 week | 58.3% | 0.091 | 0.75 | [0.21, 1.29] | 2.74 | 0.006 |
|  | 8 week | 74.7% | 0.047 | 0.32 | [-0.48, 1.12] | 0.79 | 0.432 |
|  | 12 week | 84.5% | 0.000 | 0.38 | [-0.19, 0.95] | 1.31 | 0.189 |
|  | 24 week | 84.7% | 0.000 | 1.08 | [0.37, 1.79] | 2.97 | 0.003 |
|  | 48 week | 91.3% | 0.000 | 0.56 | [-0.44, 1.55] | 1.09 | 0.275 |
|  | Overall | 82.1% | 0.000 | 0.60 | [0.37, 0.83] | 5.15 | 0.000 |
| Hepatic arterial injection | Baseline | 0.0% | 0.801 | 0.09 | [-0.10,0.28] | 0.42 | 0.673 |
|  | 24 week | 4.9% | 0.349 | 0.43 | [0.00,0.86] | 1.94 | 0.052 |
|  | Overall | 56.4% | 0.008 | 0.43 | [0.17,0.69] | 3.25 | 0.001 |
| **Cell type** |  |  |  |  |  |  |  |
| BM-MSC | Baseline | 4.6% | 0.395 | 0.08 | [-0.12, 0.28] | 0.79 | 0.428 |
|  | 2 week | 0.0% | 0.338 | 0.81 | [0.50, 1.11] | 5.21 | 0.000 |
|  | 4 week | 65.2% | 0.090 | 0.97 | [0.33, 1.60] | 2.97 | 0.003 |
|  | 12 week | 88.4% | 0.000 | 0.61 | [-0.81, 2.04] | 0.85 | 0.397 |
|  | 24 week | 74.0% | 0.009 | 0.82 | [0.09, 1.55] | 2.20 | 0.028 |
|  | 48 week | 74.6% | 0.047 | -0.63 | [-1.90,0.65] | 0.96 | 0.336 |
|  | Overall | 75.3% | 0.000 | 0.44 | [0.17, 0.71] | 3.22 | 0.001 |
| UC-MSC | Baseline | 30.1% | 0.231 | 0.27 | [-0.01, 0.55] | 1.90 | 0.057 |
|  | 4 week | 0.0% | 0.000 | 0.49 | [0.09,0.88] | 2.43 | 0.015 |
|  | 8 week | 74.7% | 0.047 | 0.32 | [-0.48,1.12] | 0.79 | 0.432 |
|  | 12 week | 82.6% | 0.001 | 0.41 | [-0.18,1.00] | 1.36 | 0.175 |
|  | 24 week | 83.1% | 0.003 | 0.87 | [0.13,1.61] | 2.30 | 0.022 |
|  | 36 week | 94.2% | 0.000 | 1.85 | [-0.42,4.11] | 1.60 | 0.110 |
|  | 48 week | 88.2% | 0.000 | 1.09 | [0.17,2.00] | 2.32 | 0.020 |
|  | Overall | 81.3% | 0.000 | -0.14 | [-0.48,0.19] | 5.20 | 0.000 |

**Supplementary Table 3.** Results of administration route and cell type of MSCs therapy on TBIL level.

|  | **Time point** | **Heterogeneity** | | **SMD** | **95%-CI** | ***Z*** | ***p*-value** |
| --- | --- | --- | --- | --- | --- | --- | --- |
|  |  | ***I2*** | ***p-value*** |  |  |  |  |
| **Administration route** |  |  |  |  |  |  |  |
| Intravenous injection | Baseline | 91.5% | 0.000 | 0.42 | [-0.29, 1.14] | 1.15 | 0.248 |
|  | 4 week | 96.8% | 0.000 | 0.95 | [-0.94, 2.84] | 0.98 | 0.326 |
|  | 12 week | 58.7% | 0.064 | -0.25 | [-0.63, 0.13] | 1.29 | 0.196 |
|  | 24 week | 85.5% | 0.000 | -0.09 | [-0.68, 0.49] | 0.31 | 0.757 |
|  | 48 week | 72.5% | 0.056 | -0.23 | [-0.87, 0.42] | 0.69 | 0.489 |
|  | Overall | 89.3% | 0.000 | 0.11 | [-0.20, 0.41] | 0.70 | 0.484 |
| Hepatic arterial injection | Baseline | 50.1% | 0.111 | -0.21 | [-0.60,0.18] | 1.05 | 0.294 |
|  | 24 week | 0.0% | 0.469 | -0.11 | [-0.52,0.31] | 0.50 | 0.616 |
|  | Overall | 8.0% | 0.368 | -0.14 | [-0.30,0.03] | 1.65 | 0.098 |
| **Cell type** |  |  |  |  |  |  |  |
| BM-MSC | Baseline | 45.2% | 0.078 | -0.16 | [-0.44, 0.12] | 1.12 | 0.263 |
|  | 2 week | 57.8% | 0.124 | -0.34 | [-0.88, 0.19] | 1.26 | 0.209 |
|  | 4 week | 83.3% | 0.003 | -0.12 | [-0.72, 0.49] | 0.38 | 0.706 |
|  | 12 week | 0.0% | 0.939 | -0.86 | [-1.41, -0.32] | 3.12 | 0.002 |
|  | 24 week | 79.0% | 0.001 | -0.18 | [-0.79, 0.43] | 0.58 | 0.563 |
|  | Overall | 65.3% | 0.000 | -0.21 | [-0.42, 0.00] | 1.98 | 0.048 |
| UC-MSC | Baseline | 96.2% | 0.000 | 0.27 | [-0.01, 0.55] | 1.41 | 0.158 |
|  | 12 week | 0.0% | 0.435 | -0.05 | [-0.27,0.17] | 0.44 | 0.659 |
|  | 24 week | 71.9% | 0.059 | 0.05 | [-0.58,0.68] | 0.16 | 0.873 |
|  | 48 week | 72.5% | 0.056 | -0.23 | [-0.87,0.42] | 0.69 | 0.489 |
|  | Overall | 92.3% | 0.000 | 0.43 | [-0.01,0.86] | 1.96 | 0.054 |
